# Peri-therapeutic multi-modal hemodynamic assessment and detection of predictors for symptomatic in-stent restenosis after percutaneous transluminal angioplasty and stenting

**DOI:** 10.3389/fneur.2023.1136847

**Published:** 2023-04-18

**Authors:** Xiaowen Song, Hancheng Qiu, Shuo Yang, Yuqi Liu, Yong Cao, Shuo Wang, Jizong Zhao

**Affiliations:** ^1^Department of Neurosurgery, Beijing Tiantan Hospital, Capital Medical University, Beijing, China; ^2^China National Clinical Research Center for Neurological Diseases, Beijing, China; ^3^Department of Vascular Neurosurgery, New Era Stroke Care and Research Institute, The PLA Rocket Force General Hospital, Beijing, China; ^4^Escope Innovation Academy, Beijing, China

**Keywords:** intracranial artery stenosis, percutaneous transluminal angioplasty and stenting, quantitative digital subtraction angiography, computational fluid dynamics, multi-modal hemodynamic

## Abstract

**Backgrounds:**

This study performed multi-modal hemodynamic analysis including quantitative color-coded digital subtraction angiography (QDSA) and computational fluid dynamics (CFD) to delineate peri-therapeutic hemodynamic changes and explore the risk factors for in-stent restenosis (ISR) and symptomatic ISR (sISR).

**Methods:**

Forty patients were retrospectively reviewed. Time to peak (TTP), full width at half maximum (FWHM), cerebral circulation time (CCT), angiographic mean transit time (aMTT), arterial stenosis index (ASI), wash-in gradient (WI), wash-out gradient (WO) and stasis index were calculated with QDSA and translesional pressure ratio (PR) and wall shear stress ratio (WSSR) were quantified from CFD analysis. These hemodynamic parameters were compared between before and after stent deployment and multivariate logistic regression model was established to detect predictors for ISR and sISR at follow-up.

**Results:**

It was found that stenting generally reduced TTP, stasis index, CCT, aMTT and translesional WSSR while significantly increased translesional PR. ASI decreased after stenting, and during the mean follow-up time of 6.48 ± 2.86 months, lower ASI (<0.636) as well as larger stasis index were corroborated to be independently associated with sISR. aMTT showed a linear correlation with CCT before and after stenting.

**Conclusion:**

PTAS not only improved cerebral circulation and blood flow perfusion but also changed local hemodynamics significantly. ASI and stasis index derived from QDSA were proved to play a prominent role in risk stratification for sISR. Multi-modal hemodynamic analysis could facilitate intraoperative real-time hemodynamic monitoring and help the determination of the end point of intervention.

## Introduction

Patients with 70–99% symptomatic intracranial artery stenosis (ICAS) showed 13.9–15.1% risk of same-territory ischemic stroke or transient ischemic attack (TIA) at 1-year and 19.8–22.7% risk of any stroke or death at 2 years, respectively, during the treatment with dual antiplatelet agents (1–6). Considering the high risk of recurrent stroke during medical management, percutaneous transluminal angiography and stenting (PTAS) could be an alternative treatment. The WEAVE (Wingspan Stent System Post Market Surveillance) /WOVEN trial (Wingspan One-year Vascular Events and Neurologic Outcomes) reported a low periprocedural complication rate (2.6%) and 1-year stroke and death rate (8.5%) (7, 8) showing PTAS was an effective approach to decrease stroke recurrence for patients with symptomatic ICAS who had not responded to aggressive medical management (9).

In-stent restenosis (ISR) after PTAS was associated with almost 80% of postprocedural ischemic stroke events (10). The 1-, 2-, and 3-year rate for symptomatic ISR (sISR) in the SAMMPRIS trial (Stenting and Aggressive Medical Management for the Prevention of Recurrent Stroke in Intracranial Stenosis) were 9.6, 11.3, and 14.0%, respectively, (11) and 10.9% in the US Wingspan Registry trial (9). Deteriorating patients’ long-term outcome, ISR represented a potential limitation of any stenting procedure. Therefore, identifying the predictors for ISR and sISR and finding out the appropriate method to determine the individualized optimal immediate angiographic performance in order to decrease ischemia recurrence and to improve prognosis needed imperative investigation.

Nowadays, more and more studies tuned to focus on the role of local hemodynamics in the initiation and development of ICAS (12, 13). Except for the commonly used computational fluid dynamics (CFD), quantitative color-coded digital subtraction angiography (QDSA) had been developed to enhance the conspicuity of various cerebrovascular diseases without using extra contrast medium or radiation and to provide quantitative information on the blood flow and temporal enhancement of the parenchyma (14). Meanwhile, the perfusion parameters, such as arrival time and time to peak (TTP), could also be assessed from the time–density curve (TDC) (15). This technique had been previously used in the analysis of perfusion changes in cerebrovascular diseases, namely carotid stenosis, carotid-cavernous fistula and moyamoya disease (12, 16), and the peritherapeutic assessment during cerebrovascular interventional surgery (17, 18).

In this retrospective study, both CFD and QDSA were adopted to perform multi-modal hemodynamic analysis in ICAS. Multiple parameters obtained from QDSA and CFD models were evaluated to understand the hemodynamic and perfusion changes before and after stent placement, to explore independent risk factors for ISR and sISR, and to delineate the correlations between hemodynamic features and angiographic perfusion changes in patients with 70–99% symptomatic severe ICAS.

## Methods

### Patients’ selection

The participants included in this study were from a single center cohort of ICAS patients treated with PTAS between January 2020 and March 2022. A total of 72 consecutive patients diagnosed with chronic ICAS in Beijing Tiantan Hospital were reviewed including clinical records and radiological data. The inclusion criteria were as follows: (1) Patients with the diagnosis of 70–99% atherosclerotic stenosis of a major intracranial artery [intracranial portion of internal carotid artery (ICA), M1 middle cerebral artery (MCA-M1), V4 vertebral artery (VA-V4) or basilar artery (BA)], as revealed by digital subtraction angiography (DSA); (2) Computed tomography perfusion (CTP) imaging showing apparent hypoperfusion in the corresponding territory of the target intracranial artery; (3) History of index ischemic stroke or transient ischemic attack (TIA) attributed to the ICAS; (4) Patients received PTAS; (5) Patients’ preoperative, postoperative and follow-up DSA examinations were available. Exclusion criteria were as follows: (1) The index ischemic stroke or TIA was attributed to non-atherosclerotic intracranial arterial stenosis such as moyamoya disease, vasculitis or dissection, restenosis within a stented artery, or tandem stenosis of extra-and intracranial arteries; (2) There was any potential cardioembolic source; (3) There was known intracranial tumor, arterio-venous malformation or aneurysm; (4) The patient received medical treatment for the index stroke/TIA as clinically indicated. Additionally, those without complete intracranial angiographic series (*n* = 25) and those whose DSA images were not of sufficient quality for radiological evaluation and quantitative analysis due to nonstandardized protocol and severe motion artifact (*n* = 7) were also excluded. Finally, a total of 40 patients were enrolled in this study. The medical records including demographic characteristics, history of common cardiovascular risk factors and initial clinical presentations were retrospectively collected and evaluated by two professional neurosurgeons.

This retrospective study was conducted in accordance with the 1964 Helsinki Declaration and its later amendments or comparable ethical standards. Ethic approval was waived by the ethics committee of Beijing Tiantan Hospital in view of the retrospective nature of the study.

### DSA acquisition and QDSA measurement

DSA were available preoperatively, postoperatively and at follow-up for all participants in this study. All DSA procedures were performed with a single C-arm fluoroscopy system. A standard angiographic method (transfemoral route) was applied in all patients using the same protocol. Image acquisition was performed *via* a 5F catheter with the tip positioned at the beginning of the ICA or VA. The 2-dimensional (2D) DSA series were acquired with a rate of 4 frames per second. For ICA image acquisition, 5 ml of contrast material was automatic injected in all series by power injector at a flow rate of 7 ml/s. For VA image acquisition, 3 ml of contrast material was automatic injected at a flow rate of 5 ml/s. The QDSA was obtained with postprocessing software (syngo iFlow, Siemens, Germany). For each manually drawn region of interest (ROI), the TDC was produced automatically by the software (19).

**Figure 1:**
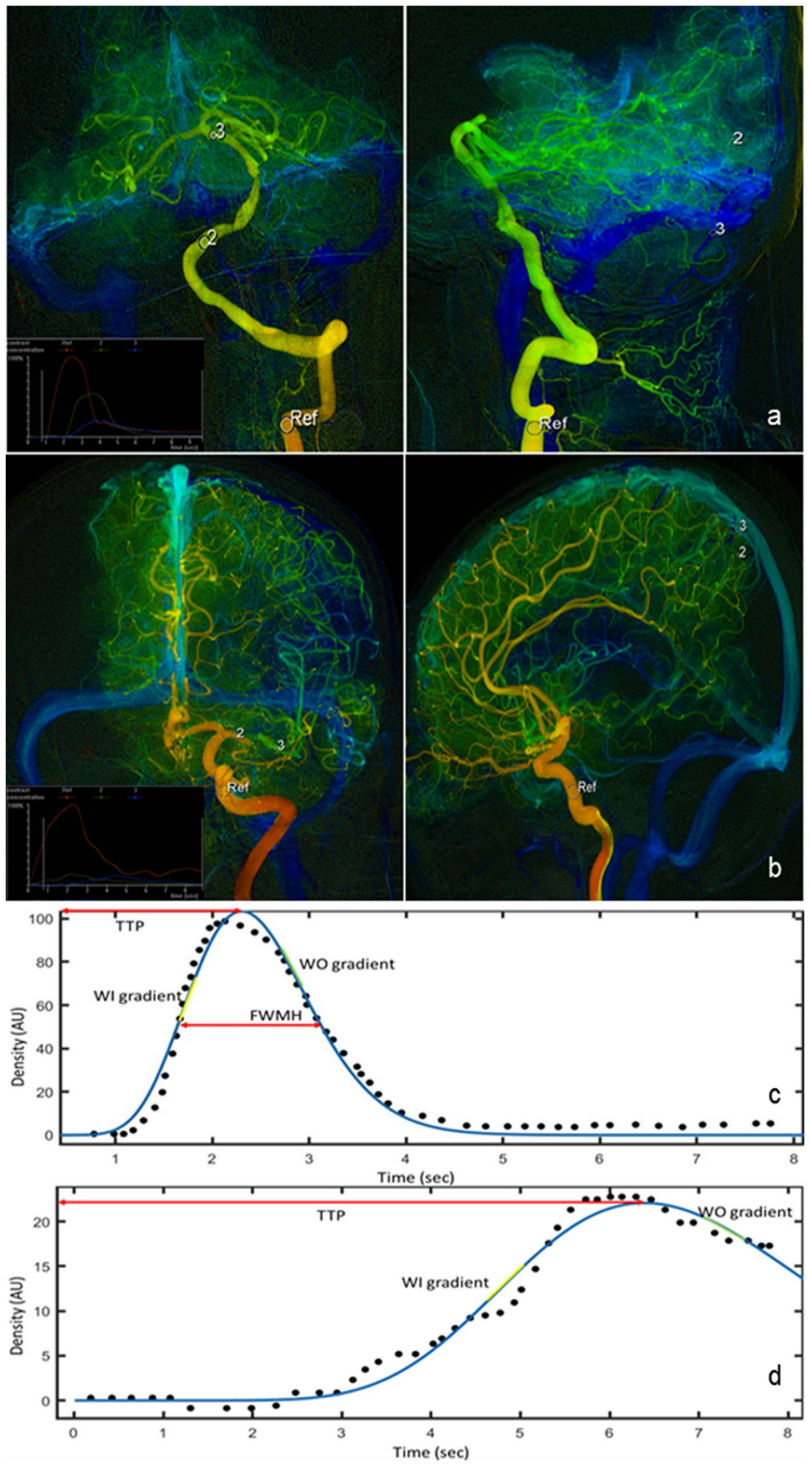
QDSA images in the PA and lateral views of lesions in posterior circulation **(A)** and lesions in anterior circulation **(B)**. Six ROIs are manually selected for quantification of perfusion parameters (white circle). The measured (black dot) and fitted (blue line) TDC of ROI in the cavernous segment of ICA **(C)** and of ROI in vein **(D)**. Hemodynamic parameters such as TTP, WI, WO, and FWHM are all derived from the fitted curve. QDSA, quantitative color-coding digital subtraction angiography; PA, posterior–anterior; ROI, region of interest; TDC, Time-density curve; ICA, internal carotid artery; TTP, time to peak; WI, washi-in gradient; WO, wash-out gradient; FWHM, full width half medium.

Six ROIs were selected for comparing hemodynamic parameters. The ROI’s diameter was the maximum diameter of the artery or vein. On the posterior–anterior (PA) view, we selected 3 ROIs: the cavernous segment of ICA for anterior circulation lesions or the V2-V3 junction for posterior circulation lesions (defined as Ref), distal to the arterial stenotic point (defined as post-stenosis) and proximal to the stenosis (defined as pre-stenosis). On the lateral view, we selected 3 ROIs: the cavernous segment of ICA for anterior circulation lesions or the V2–V3 junction for posterior circulation lesions (defined as Ref), the capillary component near the surface of the parietal lobe for anterior circulation lesions or the capillary component near the surface of the cerebellum for posterior circulation lesions (defined as capillary), and the outlet of parietal vein for anterior circulation lesions or the outlet of inferior vein of cerebellar hemisphere for posterior circulation lesions (defined as vein). Ref points in the PA view and the lateral view of the same patient were selected at the same place. The locations of these ROIs were shown in Figures 1A,B.

The TDC of selected ROIs were fitted by sum of sine or a gamma variate function as appropriate using a customized program (MATLAB, The MathWorks, United States). The following characteristics of the TDC in each ROI were used for hemodynamic analysis: time to peak (TTP), full width at half maximum (FWHM), wash-in (WO) and wash-out (WO) gradients, stasis index, and area under the curve (AUC) (Figures 1C,D). TTP was defined as the time required for the bolus to reach the maximum concentration. Relative TTP (rTTP) represented the time difference from an upstream vessel to a downstream one and was calculated between TTP of the cavernous ICA segment to the TTP of each downstream ROI (rTTP = TTP_ROI_ − TTP_Ref_). The cerebral circulation time (CCT), defined as the difference in TTP between the PV and the cavernous segment of ICA for anterior circulation stenosis or between the inferior vein of cerebellar hemisphere and the junction of V2 and V3 for posterior circulation lesions (CCT = CCT_vein_ − CCT_Ref_). FWHM was defined as the duration required for the bolus to achieve and to decline to 50% of the peak density. Angiographic mean transient time (aMTT) was used to define the full width at half maximum of the TDC derived from the capillary ROIs in the lateral views (20). The flow gradients were derived by sequentially fitting 4 consecutive temporal data points in the fitted function by using the linear least squares method. The largest and smallest slopes among all fitted linear functions were defined as the WI and WO gradients, respectively (21). Stasis index was defined as the WI gradient divided by the absolute value of the WO gradient at pre-stenosis ROI (Stasis index = WI gradient/|WO gradient|), which showed the stagnant degree of flow at the lesion (22). Arterial Stenosis Index (ASI) was defined as the ratio of the area under the curve (AUC) in the pre-stenosis ROI to the AUC in the post-stenosis ROI (ASI = AUC_pre-stenosis_/AUC_post-stenosis_) (23). Measurements were performed before and after stent deployment. Comparable measurements were assessed after stent deployment, selecting the ROIs approximately at the same locations correspondingly as those before PTAS.

The measurement of iFlow data was conducted independently by at least 2 radiologists who are with at least 5 years of clinical experiences in radiology center of our institute.

### CFD modeling and quantification of hemodynamic features

CFD analysis was performed using the patients’ 3-dimensional (3D)-DSA data before and after stenting for comparison of hemodynamic alteration caused by PTAS. 3D geometry of the arteries of interest was reconstructed from DSA source images with Mimics (Materialise, Belgium) and Magics (Materialise, Belgium), covering the ICA; MCA-M1, M2; and ACA-A1, A2 segments for cases with ICA or MCA-M1 lesion; and VA-V4, BA, PCA-P1 segments and SCA for cases with a VA-V4 or BA lesion. A mesh was then created on the vessel surface in ICEM CFD (ANSYS, United States), with the maximal element sizes of 0.1 for the inlet (s) and outlets and 0.2 for other parts of the mesh, containing at least 1 million elements in total for each case. Blood flow simulation was performed on this mesh using CFX (ANSYS, United States). The following settings and assumptions were applied in simulation of the blood flow: (1) Rigid, noncompliant walls with no-slip boundary conditions were assumed; (2) The outlet pressure was set to be 0 Pa, as we primarily focused on the relative translesional pressure change across the ICAS lesion; (3) The individualized mean flow velocity at the inlet was obtained from each patient’s transcranial Doppler; (4) Blood flow simulation was conducted in CFX-pre (ANSYS, United States) by solving the Navier–Stokes equations; convergence was achieved when the root mean square residual value reached below 10^−4^; (5) Blood was an incompressible Newtonian fluid with a constant viscosity of 0.0035 kg/(m*s) and a density of 1,060 kg/m^3^.

**Figure 2:**
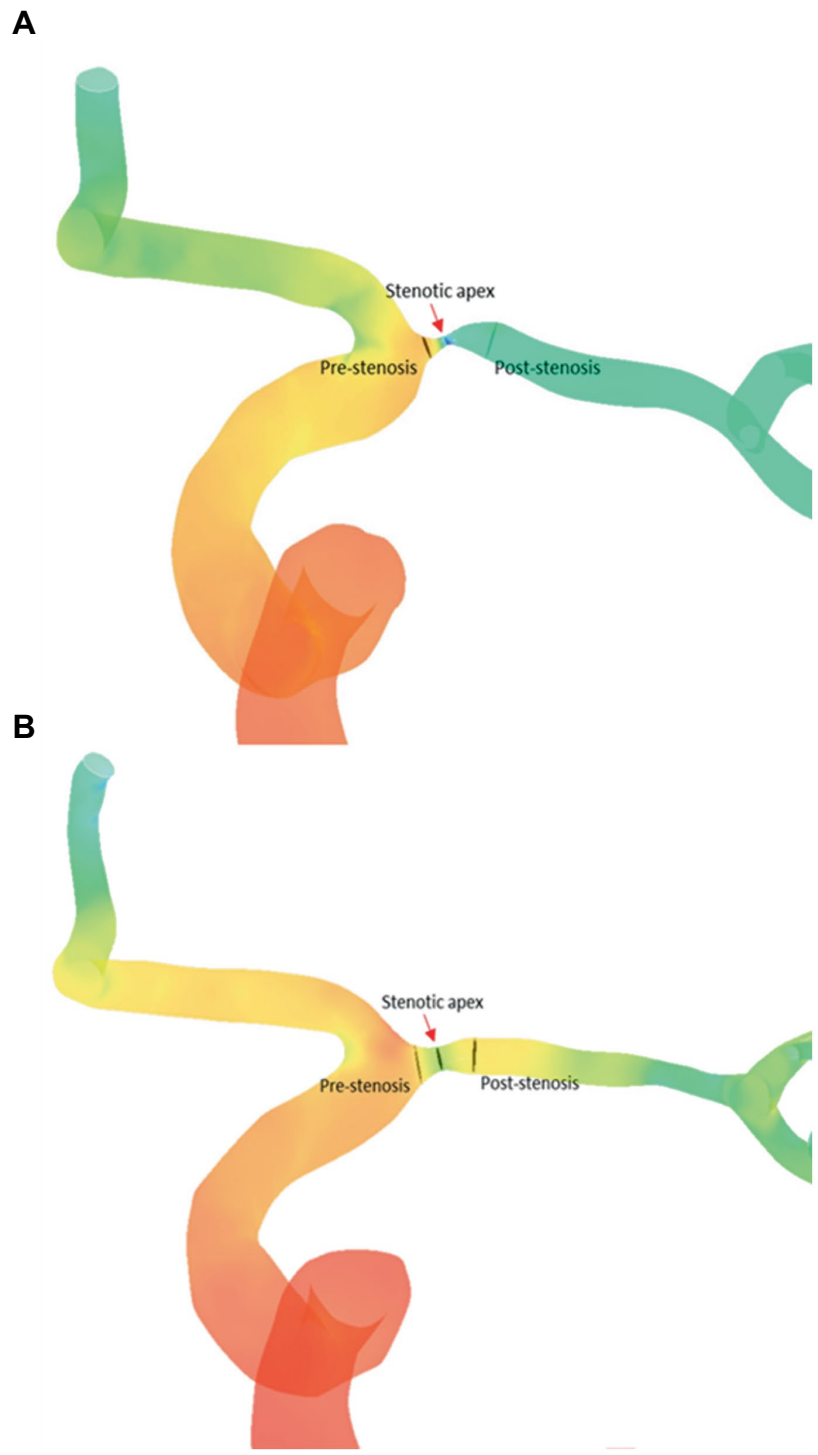
Comparation of CFD hemodynamic parameters [PR **(A)** and WSSR **(B)**] between before and after stenting. CFD, computational fluid dynamics; PR, translesional pressure ratio; WSSR, translesional wall shear stress ratio.

We quantified the relative changes of pressure and wall shear strain (WSS) across each ICAS lesion, by obtaining translesional pressure ratio (PR) and wall shear stress ratio (WSSR) on the CFD models in CFD-post (ANSYS, United States). In the pre-PTAS model, translesional PR was calculated as pressure_post-stenosis_/pressure_pre-stenosis_, whereas pressure_post-stenosis_ and pressure_pre-stenosis_ were measured at the first normal diameter distal to the ICAS lesion and at the proximal normal vessel segment, respectively. Translesional WSSR was calculated as WSS_stenotic-apex_/WSS_pre-stenosis_; WSS_stenotic-apex_ was measured at the most severely narrowed cross-section of the diseased intracranial artery referred to as the stenotic throat, and WSS_pre-stenosis_ was measured at the proximal normal vessel segment. In the post-PTAS model, these hemodynamic indices were measured at the correspondingly the same locations as in the pre-PTAS model (Figure 2).

### Endovascular procedure and angiographic and clinical follow-up

All patients had been pretreated with dual antiplatelet therapy (aspirin 300 mg and clopidogrel 75 mg daily) started at least 5 days before stenting. The PTAS procedures were successfully performed using self-expanding stents by highly experienced neurosurgeons. After stent deployment, patients were maintained on dual antiplatelet therapy for at least 3 months, which was replaced by antiplatelet monotherapy (aspirin or clopidogrel) afterward. Perioperative complications were recorded.

According to the standard follow-up regimen after PTAS at our center, initial follow-up angiography, CTP, and clinical reviews were conducted 3–12 months later after the stent deployment procedure in all included patients. Further follow-up or treatment would be performed as dictated by clinical symptoms or findings on the initial angiogram. Any adverse events, especially recurrent stroke, were recorded. MRI or CT was performed for patients with new ischemic symptoms. The follow-up DSA images were collected from the radiological database for ISR evaluation. If the luminal narrowing in the affected vessel was >50%, it would be considered restenosis. The primary outcome was defined as ISR. sISR (ISR with recurrent stroke) was considered as the secondary outcome. The modified Rankin Scale (mRS) score was obtained on admission and in the follow-up. The evaluation of mRS score was conducted by clinical neurosurgeons who had at least 5 years’ experience of clinical practice.

### Statistical analysis

The categorical variables were presented as counts (with percentages). Mean (with standard deviation) were used to describe the continuous variables with normal distribution, and median (with inter-quartile) were used to describe non-normal distribution continuous variables. All baseline variables were compared between patients with or without the primary or secondary outcome in univariate analyses, using Fisher exact test, independent-sample *t*-test or Mann–Whitney *U*-test were used as appropriate. A paired-samples *t*-test or Wilcoxon rank sum test was used to compare the differences in hemodynamic parameters pre-and post-PTAS. The Spearman test was used to evaluate the correlation between the CFD hemodynamic parameters and flow patterns acquired from QDSA. Univariate logistic regression analysis was performed on all included variables to determine their correlation with ISR and sISR after PTAS. Subsequently, the variable with *p* < 0.1 in the univariate logistic regression were included in the multivariate logistic regression analysis. C-statistics was adopted to evaluate the predictive values of the regression model. To determine the diagnostic performance of parameters in each angiogram, we performed receiver operating characteristic (ROC) analysis. The maximum area under the ROC curve was used to determine the optimal cutoff value. *p*-value <0.05 was considered to be statistically significant. Statistical analysis was performed using SPSS 22.0 (IBM, United States) and Prism (GraphPad, United States) was used to generate figures.

## Results

### Study cohort characteristics

A total of 40 patients with severe ICAS treated with PTAS were included in the study cohort (25 men, 15 women). Mean age was 56.28 years old. 7 cases were identified as ISR at follow-up (defined as ISR group) and the other 33 patients were divided into the no-ISR group. Among 7 patients diagnosed with ISR, 4 suffered from recurrent ischemic stroke and were allocated to the sISR group (*n* = 4) and the remaining patients without sISR were allocated to the no-sISR group (*n* = 36). No significant differences were observed in age, gender, stroke risk factors, or mRS on admission between ISR and no-ISR group, as well as between sISR and no-sISR group. The study cohort characteristics were summarized in Table 1. The sISR group was evaluated with significantly higher mRS at follow-up [2.00 (1.25–2.75) vs. 1.00 (0.00–1.00), *p* = 0.038] suggesting worse prognosis.

**Table 1:** Baseline demographic and clinical characteristics.

	overall (*n* = 40)	ISR (*n* = 7)	no-ISR (*n* = 33)	Value of *p*	sISR (*n* = 4)	no-sISR (*n* = 36)	Value of *p*
Age (year)	56.28 ± 8.84	57.57 ± 6.29	55.97 ± 9.41	0.672	54.75 ± 9.81	56.47 ± 8.86	0.719
Male	25 (62.50%)	3 (42.86%)	22 (66.67%)	0.392	3 (75.00%)	22 (61.11%)	>0.999
Follow-up time (m)	6.48 ± 2.86	6.71 ± 3.77	6.42 ± 2.70	0.811	6.75 ± 3.77	0.64 ± 2.81	0.843
Symptom							
Motor dysfunction	22 (55.00%)	4 (57.14%)	18 (54.55%)		3 (75.00%)	19 (52.78%)	
Sensory dysfunction	14 (35.00%)	3 (42.86%)	11 (33.33%)		2 (50.00%)	12 (33.33%)	
Aphasia	13 (32.50%)	2 (28.57%)	11 (33.33%)		1 (25.00%)	12 (33.33%)	
Headache/Dizziness	7 (17.50%)	2 (28.57%)	5 (15.15%)		1 (25.00%)	6 (16.67%)	
Dysphagia	2 (5.00%)	0	2 (6.06%)		1 (25.00%)	1 (2.78%)	
Comorbidity							
HBP	26 (65.00%)	6 (85.71%)	20 (60.61%)	0.649	3 (75.00%)	23 (63.89%)	>0.999
DM	13 (32.50%)	3 (42.86%)	10 (30.30%)	0.678	2 (50.00%)	11 (30.56%)	0.602
CAD	2 (5.00%)	0	2 (6.06%)	>0.999	0	2 (5.56%)	>0.999
Hyperlipidemia	28 (70.00%)	6 (85.71%)	22 (66.67%)	0.656	4 (100.00%)	24 (66.67%)	0.554
mRS							
On admission	2.00 (1.00–2.00)	2.00 (1.00–2.00)	2.00 (1.00–2.50)	0.306	2.00 (1.25–3.50)	2.00 (1.00–2.00)	0.645
Follow up	1.00 (0.00–2.00)	1.00 (0.00–2.00)	1.00 (0.00–1.50)	0.531	2.00 (1.25–2.75)	1.00 (0.00–1.00)	0.038*
Lesion location							
M1-MCA	19 (47.50%)	4 (57.14%)	15 (45.45%)		1 (25.00%)	18 (50.00%)	
ICA	1 (2.50%)	0	1 (3.03%)		0	1 (2.78%)	
BA	15 (37.50%)	2 (28.57%)	13 (39.39%)		3 (75.00%)	12 (33.33%)	
V4-VA	5 (12.50%)	1 (14.29%)	4 (12.12%)		0	5 (13.89%)	

ISR, in-stent restenosis; sISR, symptomatic in-stent restenosis; mRS, Modified Rankin Scale; HBP, high blood pressure; DM, diabetes mellitus; CAD, coronary artery disease; MCA, middle cerebral artery; ICA, internal carotid artery; BA, basilar artery; VA, vertebral artery.

*Statistical significance (*p*-value < 0.05).

### Peritherapeutic hemodynamic changes of PTAS

**Figure 3:**
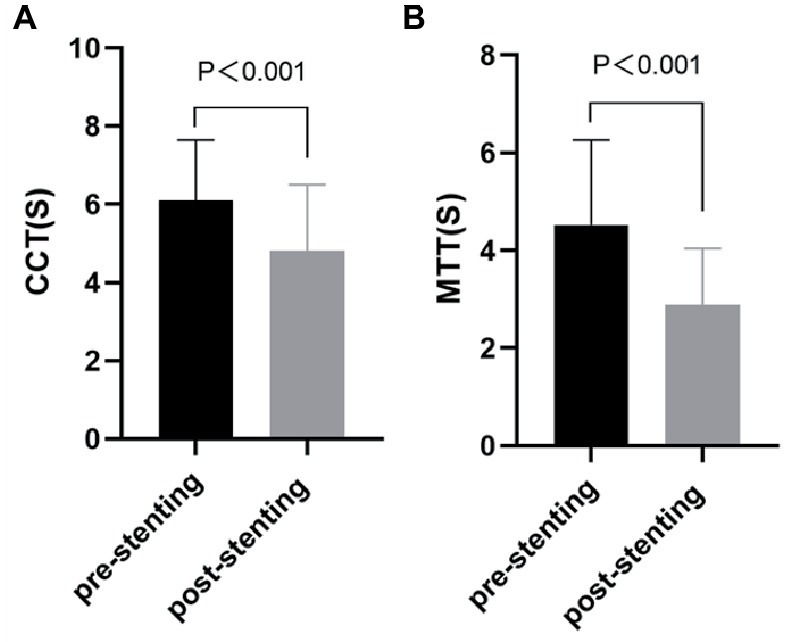
Comparation of cerebral perfusion parameters [CCT **(A)** and aMTT **(B)**] between before and after stenting. CCT, cerebral circulation time; aMTT, angiographic mean transit time.

**Figure 4:**
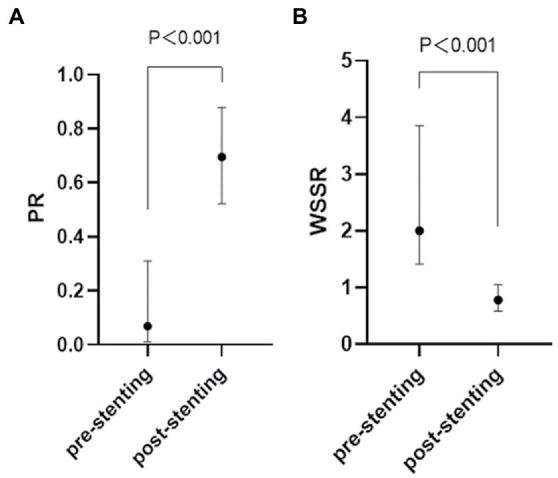
Comparation of QDSA hemodynamic parameters [ASI **(A)** and stasis index **(B)**] between before and after stenting. QDSA, quantitative digital subtraction angiography; ASI, arterial stenosis index.

After the stent-placement procedure, there was a generalized decrease in rTTP and stasis index [2.52 (1.63–4.89) vs. 1.29 (0.84–1.77), *p* < 0.001] compared with the measurements on the pre-stent angiograms indicating increased blood flow velocity (Supplementary Figure 1). According to Figure 3, stent deployment significantly shortened CCT (6.10 ± 1.55 s vs. 4.81 ± 1.70s, *p* < 0.001) and aMTT (4.52 ± 1.74 s vs. 2.88 ± 1.16 s, *p* < 0.001) revealing improved cerebral perfusion. According to Figure 4, ASI [1.27 (0.95–1.88) vs 0.92 (0.66–1.09), *p* < 0.001] and stasis index [2.52 (1.63–4.89) vs 1.29 (0.84–1.77), *p* < 0.001]decreased dramatically after stenting.

**Figure 5:**
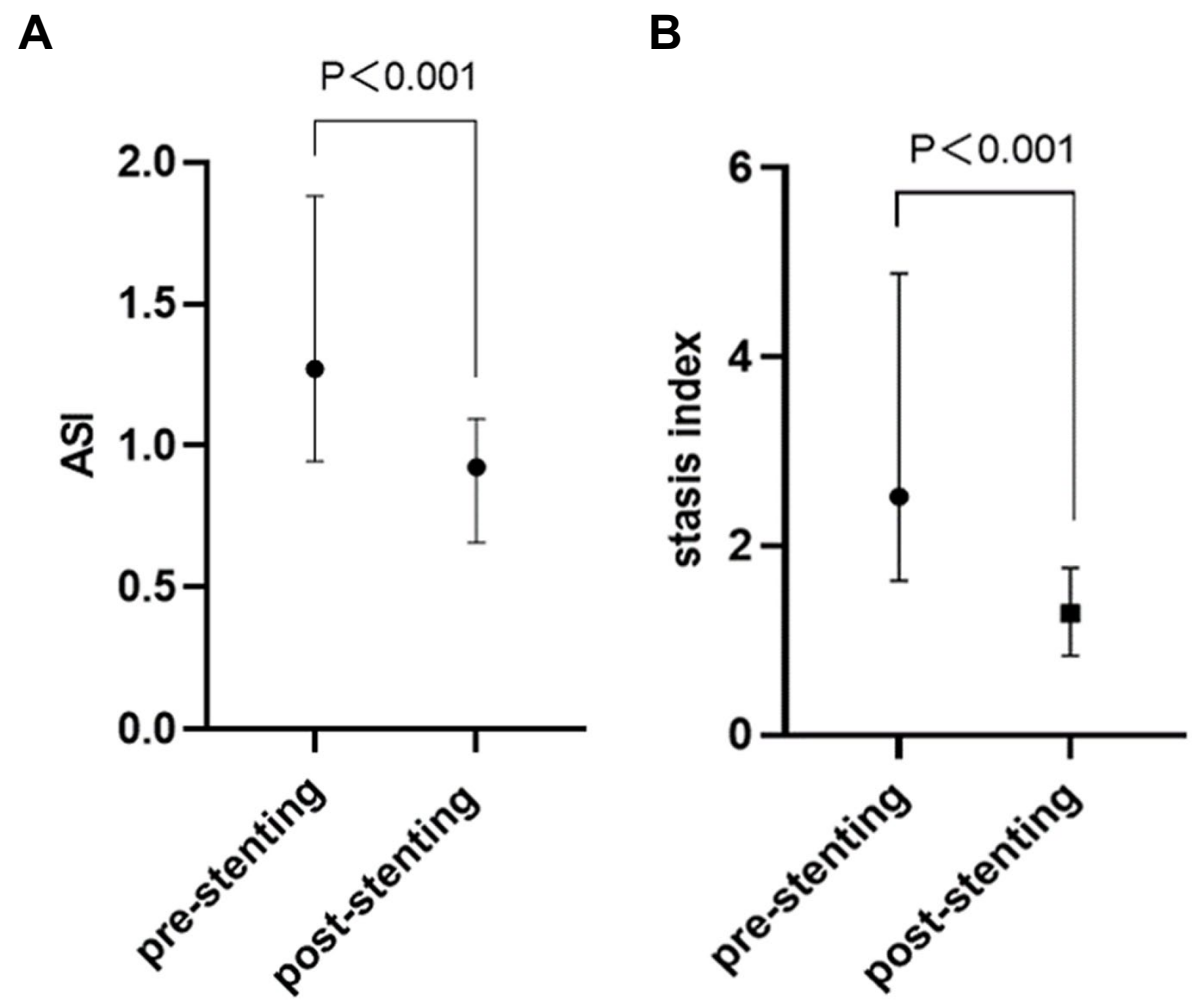
Quantification of the hemodynamic features of an ICAS lesion in the pre-stenting **(A)** and post-stenting **(B)** CFD model. ICAS, intracranial atherosclerotic stenosis; CFD, computational fluid dynamics.

The CFD hemodynamic features revealed prominent hemodynamic changes after PTAS as well. Translesional PR increased [0.07 (0.01–0.31) vs. 0.70 (0.52–0.88), *p* < 0.001], whereas WSSR decreased [2.00 (1.41–3.85) vs. 0.78 (0.58–1.05), *p* < 0.001] significantly after PTAS (Figure 5).

### Logistic regression analysis for ISR and sISR

During the mean follow-up time of 6.48 ± 2.86 months, ISR was seen in 7 cases (17.50%) and 4 of them had recurrent ischemic stroke thus being diagnosed with sISR (10.00%). Three of the four recurrent ischemic strokes were TIA and the remaining one had brainstem infarction.

**Figure 6:**
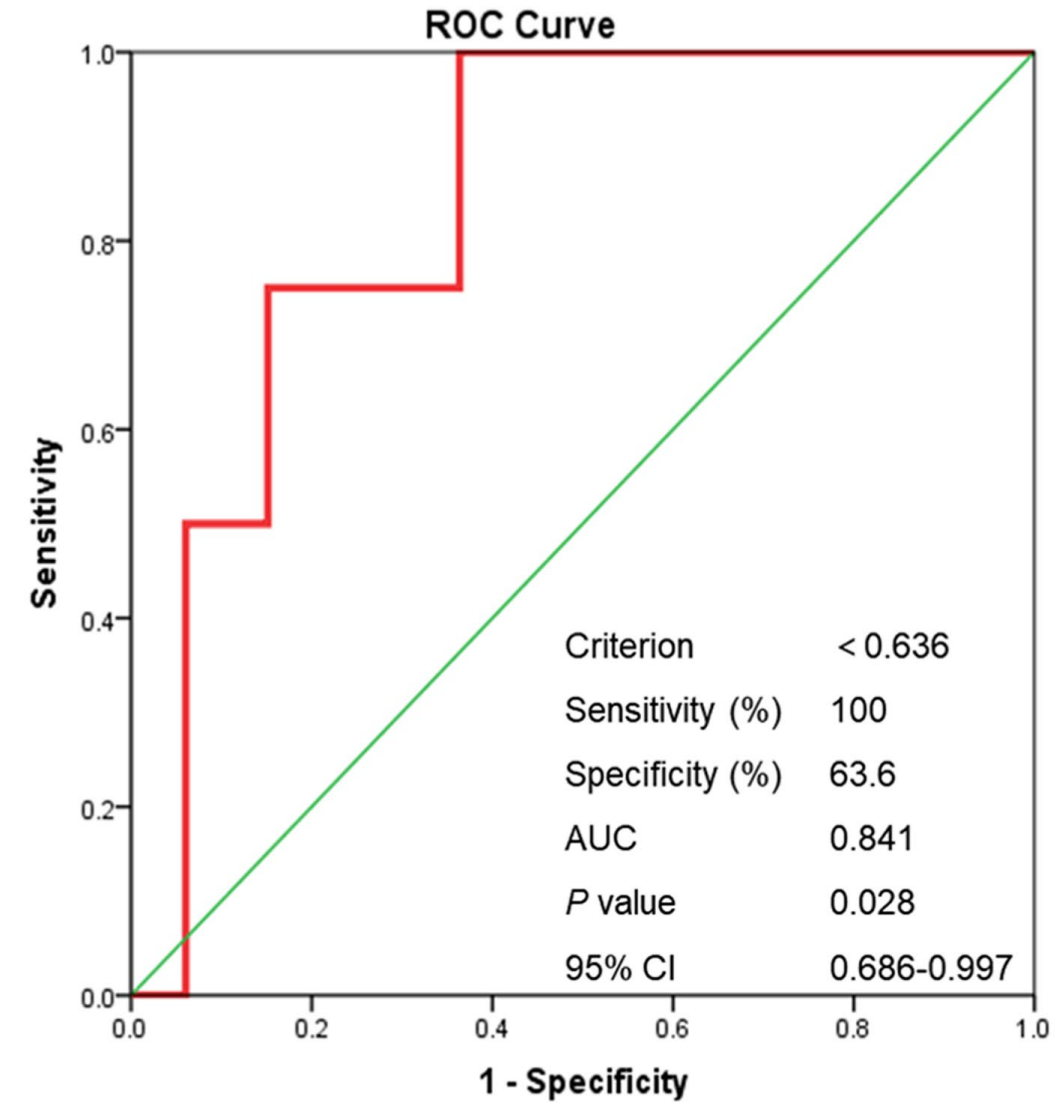
The ROC curve of post-stenting ASI. ROC curve, receiver operating characteristic curve; ASI, artery stenosis index; AUC, area under curve.

For sISR group, significantly lower ASI after stent deployment was detected when compared with no-sISR group [0.98 (0.73–1.12) vs. 0.54 (0.42–0.81), *p* < 0.001] (Supplementary Table 1). Shown as Figure 6, the AUC of the ROC was 0.841 (*p* = 0.025). A post-stenting ASI < 0.636 correctly predict symptomatic ISR in the study cohort with 100% sensitivity and 63.6% specificity.

Table 2 showed the results of the multivariable logistic analysis for the sISR. After the post-stenting ASI was stratified according to the cut-off value of 0.636, it was found that patients with lower post-stenting ASI were significantly more likely to develop sISR during follow-up after stent deployment (75.00% vs. 13.89%, *p* = 0.026). Post-stenting ASI (OR 16.80, 95% CI 1.44–195.68, *p* = 0.024) and post-stenting stasis index_pre-stenosis_ (OR 1.66, 95% CI 0.98–2.79, *p* = 0.058) were extracted from the univariate logistic analysis, and in the multivariate logistic regression, Lower post-stenting ASI (<0.636 = (OR 26.91, 95% CI 1.27–570.27, *p =* 0.035) and higher post-stenting stasis index (OR 1.84. 95% CI 1.01–3.37, *p =* 0.047) were corroborated to be independently associated with a higher risk of sISR. ROC analysis revealed substantial predictive values of this multivariable model for sISR, with the c-statistics of 0.955 (95% CI 0.887–1.000, *p* = 0.003; Supplementary Figure 2).

**Table 2:** Univariable and multivariable regression analysis for symptomatic in-stent restenosis.

	sISR (*n* = 4)	no-sISR (*n* = 36)	Univariate		Multivariate	
			Value of *p*	OR (95% CI)	Value of *p*	OR (95% CI)
Post-stenting ASI			0.024*	16.80 (1.44–195.68)	0.035*	26.91 (1.27–570.27)
Low	3 (75.00%)	5 (13.89%)				
High	1 (25.00%)	28 (77.78%)				
Post-stenting stasis index	2.54 (0.84–6.81)	1.29 (0.77–1.68)	0.058	1.66 (0.98–2.79)	0.047*	1.84 (1.01–3.37)

sISR, symptomatic in-stent restenosis; ASI, artery stenosis index.

*Statistical significance (*p*-value < 0.05).

However, when exploring the risk factors for ISR, no significant predictor was found (Supplementary Table 2).

### Correlation between various hemodynamic parameters

**Figure 7:**
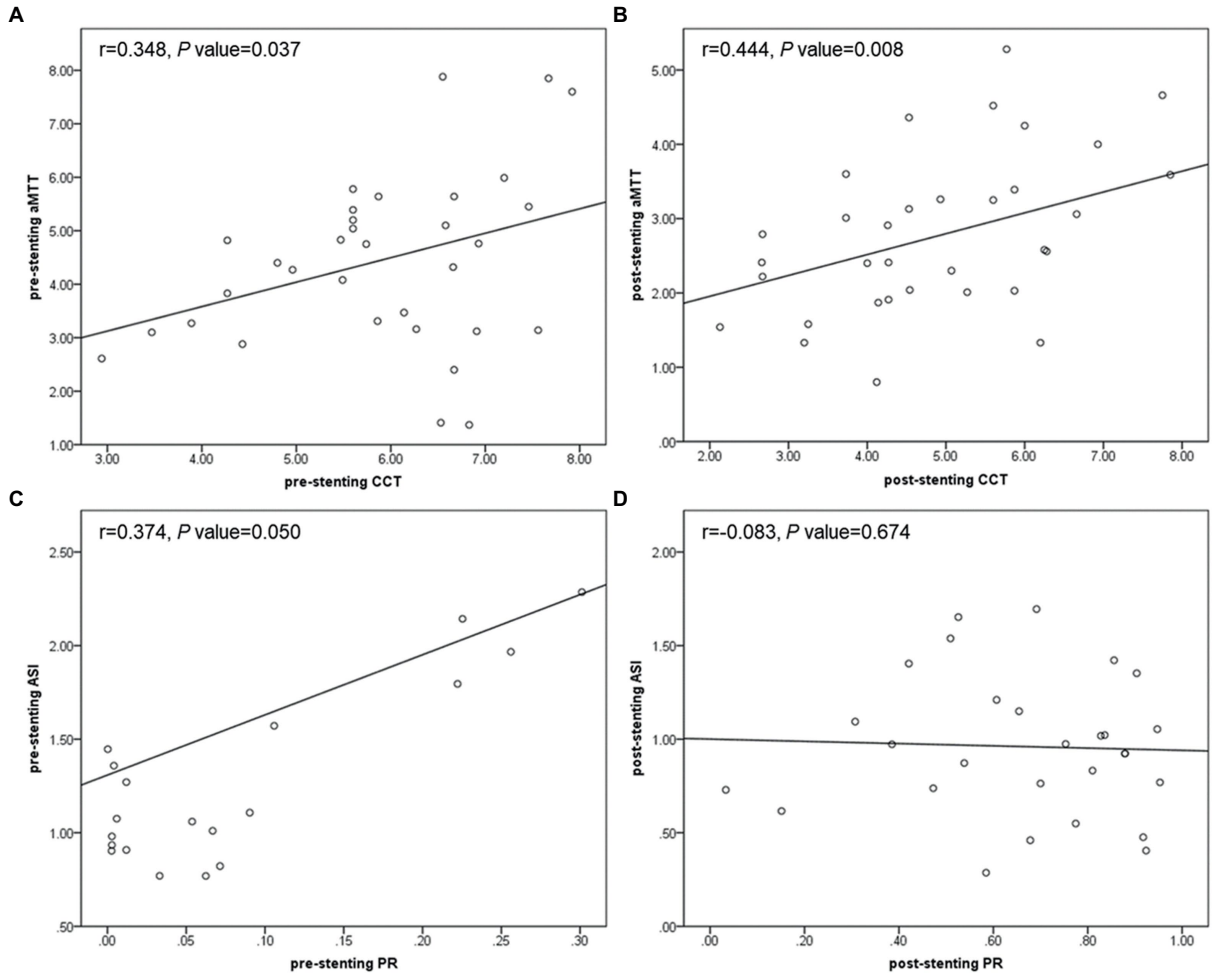
Scatter plots showing correlation between pre-stenting CCT and aMTT **(A)**, post-stenting CCT and aMTT **(B)**, pre-stenting PR and ASI **(C)**, post-stenting PR and ASI **(D)**. CCT, cerebral circulation time; aMTT, angiographic mean transit time; PR, translesional pressure ratio; ASI, artery stenosis index.

As shown in Figure 7, no matter before or after stent deployment, aMTT had a positive correlation with CCT (*r =* 0.348, *p* = 0.037; *r* = 0.444, *p* = 0.008) indicating that aMTT could become a possible alternative for CCT to reflect cerebral circulation and blood flow perfusion. PR derived from CFD seemed to be related with several hemodynamic parameters calculated from QDSA analysis. Pre-stenting ASI was linearly correlated with pre-stenting PR (*r* = 0.374, *p* = 0.050), however, after stenting, this correlation disappeared.

## Discussion

This study was among the first to perform multi-modal hemodynamic analysis including QDSA and CFD to detect the interactions between multiple parameters derived from QDSA and CFD, to delineate peri-operative hemodynamic changes and to explore the risk factors for ISR and symptomatic ISR. It was found that PTAS generally shortened rTTP, CCT, and aMTT, decreased translesional PR, ASI and stasis index while increased translesional WSSR. These findings suggests that stenting significantly improved cerebral circulation and blood flow perfusion. Furthermore, smaller post-stenting ASI (<0.636) and higher post-stenting stasis index was corroborated to be an independent risk factor for symptomatic ISR. These risk evaluations especially the cutoff value of post-stenting ASI could be of a lot help to determine the optimal angiographic performance and to reasonably control residue stenosis thus decreasing recurrent stroke and improving prognosis after PTAS.

QDSA had been shown to effectively reflect cerebral hemodynamics associated with disease severity (15, 21, 24–26). Providing an objective and immediate surrogate measurement of intravascular flow (27), this single composite color image improved the speed of manual delineation of AVMs, made the angioarchitecture and the flow characteristics of aneurysms, AVMs, fistulas, stenosis, occlusions, dissections, and tumors easier to understand and helped with the investigation of the hemodynamic parameters associated with hemorrhagic and ischemic risk (15, 22, 28–30). In addition, it facilitated real-time hemodynamic monitoring, helped the determination of the end point of intervention, and increased safety in the treatment (31–33). Examples from recent studies for patients with carotid stenosis and moyamoya disease demonstrated the feasibility of using QDSA to study parenchymal blood volume of brain and described the ability to derive various features from TDC namely TTP, inflow and outflow gradient, FWHM and CCT to evaluate cerebral circulation and blood flow perfusion (21, 34, 35). However, the role of QDSA analysis in peri-therapeutic hemodynamic evaluation and individualized risk stratification for ICAS patients treated with PTAS had not been investigated.

By plotting TDC, it was possible to indirectly extrapolate cerebral blood flow (CBF) and cerebral blood volume (CBV) by evaluating the AUC (36, 37). In patients with intracranial venous sinus stenosis, calculated as a ratio between the AUC of the ROI proximal and distal to the stenosis, VSI correctly classified hemodynamically significant stenosis (23). Similarly, in the current study, ASI, defined as a ratio between the AUC of the ROI proximal and distal to the intracranial artery stenosis, was found to be significantly decreased after stenting and was independently associated with sISR. Additionally, pre-stenting ASI was shown to be positively correlated with translesional PR (*r* = 0.374, *p* = 0.050), which further corroborated this parameter could be taken as an assessment of intracranial arterial pressure and a reliable measurement of local hemodynamics. Because this parameter established an inner control for each patient, possible confounders related to uncontrolled technical variances during DSA and contrast injection among patient sample were minimized. Therefore, with the appropriate cut-off value found in the current study, post-stenting ASI could be taken as a reliable and immediate parameter during the PTAS procedure to help with determination of the optimal angiographic performance especially the most resonable residual stenosis to decrease sISR and improve prognosis. For patients with post-stenting ASI < 0.636, they should be considered at higher risk for sISR. Shorter imaging and clinical follow-up were recommended. These findings suggested that QDSA analysis may help in risk stratification and making therapeutic decisions. Using this value derived from QDSA, the diagnostic value of DSA in the operating room could be enhanced and additional more individualized information to predict patient outcomes could be provided.

Previous studies had made some efforts to explore the risk factors for ISR and sISR, however, no definite consensus was reached. Some reported that increased residual stenosis independently predicted recurrent stroke (10, 38, 39). Conversely, a meta-analysis showed a close relationship between lower residual stenosis and higher incidence of sISR, which might be true because lower residual stenosis implied greater arterial and endothelial injury that promoted inflammation and plaque progression thus simulating ISR (40). The discrepancy might indicate that ASI might be an easily available and immediate predictor with more reliability for sISR compared with residual stenosis.

Stenosis degree had been shown to be poorly correlated with the hemodynamic status of patients with carotid stenosis. It only played an upstream role in the hemodynamics and was not reliable as the sole indicator of revascularization (25). Angiographic CCT, defined as the difference between time to peak from the cavernous portion of the ICA to the PV in lateral view, was considered as a good surrogate hemodynamic marker for demonstrating cerebral hypoperfusion and was proved to be correlated with CT and MR perfusion parameters such as CBF, MTT and Tmax in patients with carotid stenosis (32, 35, 37, 41–43). Additionally, this parameter had also been reported to be well correlated with cerebrovascular reserve (37, 44, 45). Previous studies had reported that prolonged CCT more accurately reflects the symptomatic status of patients with carotid stenosis and MCA stenosis treated with drugs than the stenosis degree (25, 46). However, this parameter was only adopted for anterior circulation stenosis. In the current study, we made a breakthrough to use this parameter in posterior circulation stenosis creatively and defined it as difference in TTP between the inferior vein of cerebellar hemisphere and the junction of V2 and V3. Consistent with previous studies, we found that CCT was significantly shortened by PTAS revealing improved cerebral circulation (6.10 ± 1.55 s vs. 4.81 ± 1.70s, *p* < 0.001). However, it failed to identify individuals at high risk of ISR or sISR during follow-up. A possible mechanism of CCT being less predictive and meaningful for ICAS patients might be the autoregulation of the brain occurring at the capillary level characterized by the development of collateral flow followed by dilatation of the vessel itself, which might have somehow neutralized the benefits of increased upstream arterial flow after stent placement (47–49).

Moreover, in order to further reflect cerebral perfusion, Han-Jui Lee et al. applied the method of independent component analysis to extract the capillary component from DSA images and introduced aMTT which defined as the full width at half maximum of the TDC derived from the capillary component (20). This variable measured the duration of contrast-containing blood flow remained in the capillary, the smallest vasculature identifiable and conceptually closest to brain parenchyma in DSA imaging. Likely, in this study, we measured aMTT at the capillary component near the surface of cerebral cortex to free researchers from the trouble applying the independent component analysis method. It was found that aMTT was significantly shorted by PTAS (4.52 ± 1.74 s vs. 2.88 ± 1.16 s, *p* < 0.001) and showed positive linear correlation no matter before (*r* = 0.348, *p* = 0.037) or after stenting (*r* = 0.444, *p* = 0.008) implying its ability to be considered as a reliable alternative and supplement to CCT, and its feasibility to provide all-round and multi-perspective evaluations of cerebral perfusion.

Except for post-stenting ASI, larger stasis index at the stenosis was also suggested to be independently associated with sISR in the current study. The stasis index was proposed and defined as the ratio of inflow gradient to outflow gradient by Lin et al. and might serve as a surrogate for intraluminal pressure imaging (22). This value was theoretically not affected by factors such as contrast medium concentration, injection delay and frame rate settings. A large stasis index might be caused by increased inflow and decreased outflow, indicating stagnant blood flow. Larger venous stasis index indicated impaired venous drainage and was reported to be associated with BAVM hemorrhage (22, 29, 30) and predicts obliteration after Gamma Knife radiosurgery (31). For severe ICAS, we found that PTAS significantly decreased stasis index showing improved cerebral circulation. Similar to BAVM, impaired outflow of the stenosis lesion after PTAS demonstrated by larger post-stenting stasis index suggesting relatively stagnant flow at the stenosis was predictive for stroke recurrence.

This retrospective study was mainly limited by its small sample size and measurement errors. Firstly, the relatively small cohort reduced the power to detect meaningful differences between groups. Nowadays the best recommendation for ICAS was medical treatment and restricted the popularization of PTAS, which might attributed to the small sample size. Secondly, DSA was basically 2D imaging. Overlapping and collapsing of vasculatures as well as artifacts of the skull on DSA might lead to inaccurate peak time measurements in the ROI. In order to minimize the measurement errors, we adopted the relative value of TTP at each ROIs. Further, only simplified CFD models with the diseased artery and adjacent arteries were reconstructed, and although individualized inlet velocity was adopted, uniform outlet conditions and blood properties were still used. In order to offset the confounders, relative rather than absolute values in the current study were adopted for analysis. Additionally, during angiography, intra-arterial injections of contrast were mixed with the physiologic blood flow without contrast from other vascular territories, and therefore, the measured TDC of the contrast was distorted away from the physiologic waveform (37). In order to make the quantification of TDC more accurate, the standardized fitted TDC was calculated in MATLAB. In future studies, more advanced CFD models adopting patient-specific boundary conditions such as the proximal and distal pressure measured by the pressure wire may provide more information about the general and local hemodynamics. And furture investigations with larger sample size and more advanced test methods such as 4D flow MRI could be more promising and instructive.

## Conclusion

In conclusion, this was the first study that combined QDSA and CFD to evaluate the state of the peri-therapeutic hemodynamic condition and investigate the prognostic factors for ISR and sISR. PTAS was able to greatly change local hemodynamics and improve cerebral circulation as well as the blood flow perfusion in ICAS. Lower ASI and larger stasis index tended to be independently associated with sISR. These findings led to consideration of whether multi-modal hemodynamic analysis could be used in ICAS to help identify high risk of sISR during the PTAS procedure, better define prognosis and assess the optimal treatment decision. Post-stenting ASI < 0.636 might thus serve as an objective and immediate angiographic parameter for risk stratification in ICAS patients with PTAS. Future studies with larger sample sizes were required for further verification and more profound investigation.

## Data availability statement

The raw data supporting the conclusions of this article will be made available by the authors, without undue reservation.

## Ethics statement

The studies involving human participants were reviewed and approved by the ethics committee of Beijing Tiantan Hospital. The patients/participants provided their written informed consent to participate in this study.

## Author contributions

XS, HQ, SY, YL, YC, SW, and JZ contributed to the study conception and design. The acquisition, analysis, or interpretation of data were performed by XS, SY, and YL. The first draft was written by XS. HQ, YC, and SW involved in conception and design of the work. Revision and final approval of the draft was performed by JZ. All authors contributed to the article and approved the submitted version.

## Conflict of interest

The authors declare that the research was conducted in the absence of any commercial or financial relationships that could be construed as a potential conflict of interest.

## Publisher’s note

All claims expressed in this article are solely those of the authors and do not necessarily represent those of their affiliated organizations, or those of the publisher, the editors and the reviewers. Any product that may be evaluated in this article, or claim that may be made by its manufacturer, is not guaranteed or endorsed by the publisher.
