# Supplementary material for: Peri-therapeutic multi-modal hemodynamic assessment and detection of predictors for symptomatic in-stent restenosis after percutaneous transluminal angioplasty and stenting
Source: Front Neurol. 2023 Apr 18;14:1136847. doi: 10.3389/fneur.2023.1136847 (PMC10151536; doi:10.3389/fneur.2023.1136847)
Supplement: Supplementary file 1 [file Table_1.docx]

Supplementary table 1 Comparation of hemodynamic parameters between sISR and no-sISR group

|  | Overall (n=40) | sISR (n=4) | no-sISR (n=36) | *P* value | Univariate | |
| --- | --- | --- | --- | --- | --- | --- |
|  |  |  |  |  | *P* value | OR (95% CI) |
| pre-stenting PR | 0.07 (0.01-0.31) | 0.17 (0.01-0.48) | 0.07 (0.01-0.27) | 0.930 | 0.665 | 2.89 (0.02-352.17) |
| pre-stenting WSSR | 2.00 (1.41-3.85) | 1.97 (1.50-3.92) | 2.00 (1.41-3.85) | 0.976 | 0.789 | 0.92 (0.51-1.68) |
| pre-stenting ASI | 1.27 (0.95-1.88) | 1.04 (0.99-1.29) | 1.28 (0.92-1.98) | 0.555 | 0.366 | 0.29 (0.02-4.24) |
| pre-stenting rTTP_pre-stenosis_, s | 0.61±0.54 | 0.80±0.58 | 0.59±0.54 | 0.485 | 0.478 | 1.92 (0.32-11.60) |
| pre-stenting rTTP_post-stenosis_, s | 0.99±0.65 | 1.13±0.67 | 0.97±0.66 | 0.647 | 0.639 | 1.45 (0.31-6.89) |
| pre-stenting rTTP_capillary_, s | 2.10±0.96 | 2.62±0.71 | 2.04±0.98 | 0.261 | 0.264 | 1.90 (0.62-5.84) |
| pre-stenting rTTP_vein_, s | 6.03±1.59 | 6.57±1.79 | 5.96±1.58 | 0.479 | 0.470 | 1.27 (0.66-2.43) |
| pre-stenting CCT, s | 6.10±1.55 | 6.57±1.79 | 6.04±1.54 | 0.528 | 0.518 | 1.25 (0.64-2.42) |
| pre-stenting aMTT, s | 4.52±1.74 | 5.60±2.21 | 4.39±1.67 | 0.196 | 0.202 | 1.48 (0.81-2.70) |
| pre-stenting stasis index | 2.52 (1.63-4.89) | 3.33 (1.68-5.55) | 2.52 (1.58-4.89) | 0.811 | 0.833 | 1.05 (0.65-1.70) |
| post-stenting PR | 0.70 (0.52-0.88) | 0.61 (0.25-0.86) | 0.73 (0.52-0.88) | 0.576 | 0.401 | 0.17 (0.00-10.45) |
| post-stenting WSSR | 0.78 (0.58-1.05) | 0.58 (0.44-0.75) | 0.85 (0.59-1.17) | 0.139 | 0.233 | 0.14 (0.01-3.63) |
| post-stenting ASI | 0.92 (0.66-1.09) | 0.54 (0.42-0.81) | 0.97 (0.73-1.12) | 0.025^*^ | 0.060 | 0.01 (0.00-1.19) |
| post-stenting rTTP_pre-stenosis_, s | 0.49±0.46 | 0.60±0.34 | 0.48±0.48 | 0.614 | 0.606 | 1.70 (0.23-12.91) |
| post-stenting rTTP_post-stenosis_, s | 0.57±0.46 | 0.73±0.26 | 0.55±0.47 | 0.454 | 0.449 | 2.15 (0.30-15.68) |
| post-stenting rTTP_capillary_, s | 1.33±0.63 | 1.53±0.15 | 1.31±0.66 | 0.110 | 0.504 | 1.72 (0.35-8.37) |
| post-stenting rTTP_vein_, s | 4.81±1.70 | 5.37±1.13 | 4.75±1.75 | 0.493 | 0.484 | 1.25 (0.67-2.30) |
| post-stenting CCT, s | 4.81±1.70 | 5.37±1.13 | 4.75±1.75 | 0.493 | 0.484 | 1.25 (0.67-2.30) |
| post-stenting aMTT, s | 2.88±1.16 | 3.03±1.07 | 2.86±1.19 | 0.790 | 0.783 | 1.13 (0.47-2.74) |
| post-stenting stasis index | 1.29 (0.84-1.77) | 2.54 (0.84-6.81) | 1.29 (0.77-1.68) | 0.472 | 0.058 | 1.66 (0.99-2.79) |

sISR: symptomatic in-stent restenosis; PR: translesional pressure ratio; WSSR: translesional wall shear stress; ASI: artery stenosis index; rTTP: relative time to peak; CCT: cerebral circulation time; aMTT: angiographic mean transit time;

^*^Statistical significance (*P* value＜0.05)
