# Supplementary material for: Peri-therapeutic multi-modal hemodynamic assessment and detection of predictors for symptomatic in-stent restenosis after percutaneous transluminal angioplasty and stenting
Source: Front Neurol. 2023 Apr 18;14:1136847. doi: 10.3389/fneur.2023.1136847 (PMC10151536; doi:10.3389/fneur.2023.1136847)
Supplement: Supplementary file 2 [file Table_2.docx]

Supplementary table 2 Comparation of hemodynamic parameters between ISR and no-ISR group

|  | Overall (n=40) | ISR (n=7) | no-ISR (n=33) | *P* value | Univariate | |
| --- | --- | --- | --- | --- | --- | --- |
|  |  |  |  |  | *P* value | OR (95% CI) |
| pre-stenting PR | 0.07 (0.01-0.31) | 0.01 (0.00-0.17) | 0.09 (0.03-0.21) | 0.360 | 0.332 | 0.08 (0.00-13.48) |
| pre-stenting WSSR | 2.00 (1.41-3.85) | 1.93 (1.64-3.24) | 2.01 (1.64-5.97) | 0.158 | 0.233 | 0.67 (0.35-1.29) |
| pre-stenting ASI | 1.27 (0.95-1.88) | 1.08 (1.03-1.22) | 1.45 (1.01-2.04) | 0.608 | 0.541 | 0.76 (0.32-1.81) |
| pre-stenting rTTP_pre-stenosis_, s | 0.61±0.54 | 0.52±0.54 | 0.77±0.53 | 0.625 | 0.617 | 0.66 (0.13-3.35) |
| pre-stenting rTTP_post-stenosis_, s | 0.99±0.65 | 0.72±0.63 | 1.04±0.60 | 0.239 | 0.240 | 0.41 (0.09-1.81) |
| pre-stenting rTTP_capillary_, s | 2.10±0.96 | 1.90±0.98 | 2.14±0.97 | 0.555 | 0.545 | 0.76 (0.31-1.86) |
| pre-stenting rTTP_vein_, s | 6.03±1.59 | 5.57±2.24 | 6.40±1.34 | 0.553 | 0.406 | 0.79 (0.46-1.37) |
| pre-stenting CCT, s | 6.10±1.55 | 5.57±2.24 | 6.40±1.34 | 0.491 | 0.323 | 0.75 (0.42-1.33) |
| pre-stenting aMTT, s | 4.52±1.74 | 5.22±2.20 | 4.26±1.51 | 0.245 | 0.243 | 1.33 (0.82-2.15) |
| pre-stenting stasis index | 2.52 (1.63-4.89) | 4.85 (3.24-5.32) | 2.41 (1.45-3.95) | 0.728 | 0.608 | 0.90 (0.59-1.37) |
| post-stenting PR | 0.70 (0.52-0.88) | 0.54 (0.34-0.61) | 0.77 (0.54-0.89) | 0.532 | 0.454 | 0.27 (0.01-8.33) |
| post-stenting WSSR | 0.78 (0.58-1.05) | 0.46 (0.45-0.58) | 0.84 (0.65-0.97) | 0.335 | 0.520 | 0.58 (0.11-3.06) |
| post-stenting ASI | 0.92 (0.66-1.09) | 0.62 (0.54-0.74) | 0.92 (0.75-1.01) | 0.608 | 0.646 | 0.57 (0.05-6.43) |
| post-stenting rTTP_pre-stenosis_, s | 0.49±0.46 | 0.45±0.29 | 0.56±051 | 0.830 | 0.825 | 0.81 (0.13-5.25) |
| post-stenting rTTP_post-stenosis_, s | 0.57±0.46 | 0.53±0.31 | 0.64±051 | 0.811 | 0.805 | 0.79 (0.12-5.22) |
| post-stenting rTTP_capillary_, s | 1.33±0.63 | 1.11±0.54 | 1.35±0.68 | 0.306 | 0.302 | 0.45 (0.10-2.05) |
| post-stenting rTTP_vein_, s | 4.81±1.70 | 4.15±1.67 | 4.98±1.51 | 0.259 | 0.256 | 0.73 (0.43-1.25) |
| post-stenting CCT, s | 4.81±1.70 | 4.15±1.67 | 4.98±1.51 | 0.259 | 0.256 | 0.73 (0.43-1.25) |
| post-stenting aMTT, s | 2.88±1.16 | 3.18±1.48 | 2.73±1.03 | 0.455 | 0.445 | 1.32 (0.65-2.65) |
| post-stenting stasis index | 1.29 (0.84-1.77) | 4.22 (2.52-5.95) | 1.04 (0.92-1.47) | 0.917 | 0.250 | 1.30 (0.83-2.04) |

ISR: in-stent restenosis; PR: translesional pressure ratio; WSSR: translesional wall shear stress; ASI: artery stenosis index; rTTP: relative time to peak; CCT: cerebral circulation time; aMTT: angiographic mean transit time
