# Supplementary figures and images for: Peri-therapeutic multi-modal hemodynamic assessment and detection of predictors for symptomatic in-stent restenosis after percutaneous transluminal angioplasty and stenting
Source: Front Neurol. 2023 Apr 18;14:1136847. doi: 10.3389/fneur.2023.1136847 (PMC10151536; doi:10.3389/fneur.2023.1136847)

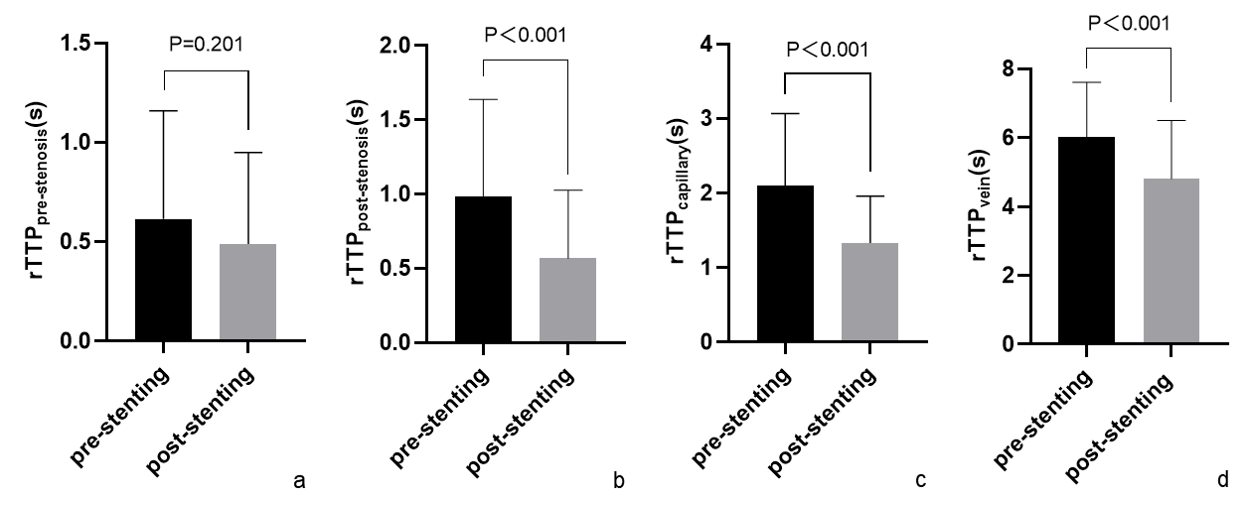

Supplement: Supplementary file 3 [file Image_1.tif]

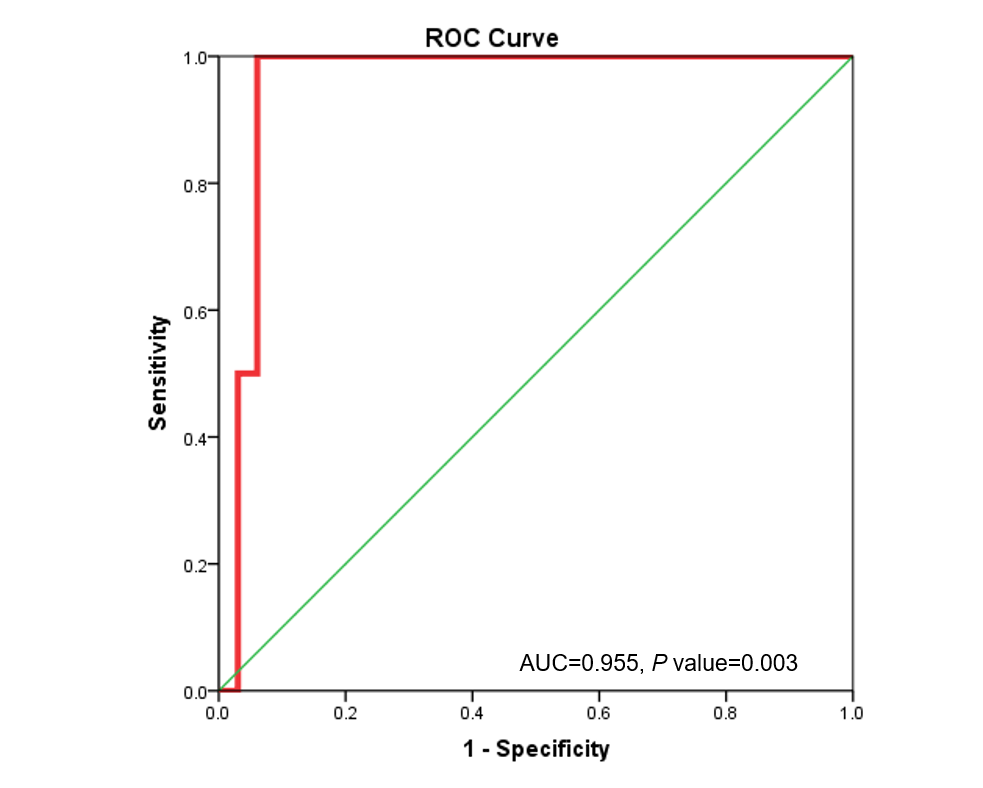

Supplement: Supplementary file 4 [file Image_2.tif]
